# Development of novel Ti-Mo-Mn alloys for biomedical applications

**DOI:** 10.1038/s41598-020-62865-4

**Published:** 2020-04-14

**Authors:** Mariana Luna Lourenço, Giovana Collombaro Cardoso, Karolyne dos Santos Jorge Sousa, Tatiani Ayako Goto Donato, Fenelon Martinho Lima Pontes, Carlos Roberto Grandini

**Affiliations:** 1 UNESP - Univ Estadual Paulista, Laboratório de Anelasticidade e Biomateriais, 17.033-360 Bauru, SP Brazil; 2 IBTN-Br – Institute of Biomaterials, Tribocorrosion and Nanomedicine – Brazilian Branch, 17.033-360 Bauru, SP Brazil; 3 UNESP – Univ Estadual Paulista, Departamento de Química, 17.033-360 Bauru, SP Brazil

**Keywords:** Cell adhesion, Biomedical materials

## Abstract

Due to excellent biocompatibility and corrosion resistance, the application of titanium alloys in orthopedic and dental implants has been increasing since the 1970s. However, the elasticity of these alloys as measured by their Young’s modulus is still about two to four times higher than that of human cortical bone. The most widely used titanium alloy for biomedical applications is Ti-6Al-4V, however, previous studies have shown that the vanadium used in this alloy causes allergic reactions in human tissue and aluminum, also used in the alloy, has been associated with neurological disorders. To solve this problem, new titanium alloys without the presence of these elements and with the addition of different elements, usually beta-stabilizers, are being developed. Manganese is a strong candidate as an alloying element for the development of new beta-type titanium alloys, due to its abundance and low cytotoxicity. In this study, Ti-10Mo-5Mn, Ti-15Mo-2.5Mn and Ti-15Mo-5Mn alloys were prepared in an arc furnace, which resulted in an alloy structure clearly showing the predominance of the beta phase with a body-centered cubic crystalline structure. The observed microstructure confirmed the results on the structural characterization of alloys. Measurement of the indirect cytotoxicity of the alloys showed that the extracts of the studied alloys are not cytotoxic for fibroblastic cells.

## Introduction

Metallic biomaterials are used in about 70–80% of implants, so the study of new materials with properties more adequate for such uses is of utmost importance. Titanium alloys are frequently used for biomedical purposes such as dental and orthopedic implants and other devices such as plates and screws because they have a favorable set of properties including good biocompatibility, resistance to corrosion and wear, excellent mechanical properties, and good osseointegration^1,2^. The properties of other materials used in the biomedical area, such as 316 L stainless steel, Co-Cr-Mo alloys^3–6^, and the Ti-6Al-4V ELI alloy, are not as good as those of titanium alloys. Ti-6Al-4V ELI, for example, contains aluminum and vanadium, elements that can cause problems in long term uses. Aluminum ions have been shown to cause an allergic reaction in human tissues and vanadium has been associated with neurological disorders (such as Alzheimer’s disease)^7–9^. Therefore, new alloys without the presence of cytotoxic elements are currently being sought.

Recent studies have focused on β-type alloys because they have a low modulus of elasticity, which allows them to avoid the stress-shielding effect. The manufacture of these alloys uses β-stabilizers and non-cytotoxic elements such as molybdenum, tantalum, niobium, zirconium, and manganese^10–12^. The alloys used today have an elasticity modulus three to four times greater than that of human bone, which results in the causing of pain to the patient and increases the risk of implant failure. This is due to the implant receiving all the mechanical load, which causes wearing and decreasing bone density thereby leaving the bones fragile, in an effect known as stress shielding^13,14^.

The molybdenum equivalent theory was used to choose the concentration of the alloying elements to stabilize the β phase. Molybdenum is one of the main β stabilizing elements and, from 10% by weight, this phase is already reached^15^. Thus, an equation was arrived at that relates the equivalent percentage of molybdenum to other elements, indicators of β phase stabilization.

$${[Mo]}_{eq}=[Mo]+[Ta]/{5}+[Nb]/{3.6}+[W]/{2.5}+[V]/{1.5}+{1.25}[Cr]+{1.25}[Ni]+{1.7}[Mn]+{1.7}[Co]+{2.25}[Fe]$$

where: [*x*] is the concentration in percentage by weight of the referring element.

Using *[Mo]*_*eq*_ for the Ti-10Mo-5Mn, Ti-15Mo-2.5Mn and Ti-15Mo-5Mn alloys obtained 18.5 wt.%, 19.3 wt.% and 23.5 wt.% respectively.

Hong and collaborators studies with metastable β-type Ti–Cr-(Mn) cast alloys show that with 13.04 wt.% *[Mo]*_*eq*_, only the Beta phase is present^16^.

Molybdenum is a strong β-stabilizer and is regarded as the most suitable β-stabilizer element because it is able to stabilize β-phase this phase with a low solute concentration. This is important because it is a refractory metal with a high melting point. So, the addition of this element to Ti increases the melting point making the processing of the material very difficult, provides low modulus of elasticity and flexural strength^17^. Binary Ti-Mo alloys with up to 20% molybdenum have been the subject of several studies due to their having good mechanical properties and corrosion resistance, β phase predominance and non-cytotoxic behavior^1,18–21^. Moreover, the Ti-Mo binary system is considered suitable for use as orthopedic implants, which was standardized by ASTM for this type of application (ASMT 2008).

Manganese is also a low-cost, highly available element and decreases β-transus temperature that can be associated with titanium, reduces the modulus of elasticity, improves ductility and increases hardness, with desirable values for use as a biomaterial. It is one of the essential elements of the human body, essential for skeletal growth and development, which plays an important role in osteogeneses and bone resorption^10,22^, so manganese is less cytotoxic than aluminum and vanadium and up to 18% wt., its cytotoxicity is compared with Ti-cp^16,20,23^.

Fernandes Santos and collaborators studied the system of ternary alloys of the Ti-Mo-Mn system, varying the Mo between 3 and 4% and Mn between 5 and 6% by weight evaluating the microstructure, Vickers microhardness, modulus of elasticity and other mechanical properties in addition to Corrosion resistance where only the presence of the β phase was found. According to the literature, titanium alloys containing Mn are produced using the cold crucible levitation melting (CCLM) and metal injection molding (MIM) technique where the boiling point of Mn is not reached, producing the alloys according to the desired compensation^10,23^.

This paper shows the preparation and characterization of a set of novel β-Ti alloys containing molybdenum and manganese, for applications as a biomaterial.

## Results

Table 1 present the chemical composition of the samples, whose values are very close to the nominal (stoichiometric) values. The energy dispersive spectroscopy (EDS) spectra of the elements (not showed here) show peaks only for the alloy elements (titanium, molybdenum, and manganese), and not for other metallic elements, that is they do not show metallic contamination, corroborating the chemical composition result obtained by inductively coupled plasma optical emission spectrometry (ICP-OES).

**Table 1:** Chemical analysis of Ti-Mo-Mn alloys.

Element	Ti-10Mo-5Mn (wt %)	Ti-15Mo-2.5Mn (wt %)	Ti-15Mo-5Mn (wt %)
Al	0.03	0.03	0.04
Cr	0.03	0.008	0.008
Cu	0.007	0.006	0.003
Fe	0.09	0.09	0.09
Hf	0.08	0.10	0.13
Ni	0.01	0.004	0.004
Si	0.03	0.02	0.013
Ta	<0.05	<0.05	<0.05
Zr	<0.005	<0.005	<0.005
Mn	5.0	1.9	4.2
Mo	9.5	13.9	14.3
O	0.178	0.206	0.162
N	0.006	0.007	0.009
Ti	Balance	Balance	Balance

**Figure 1:**
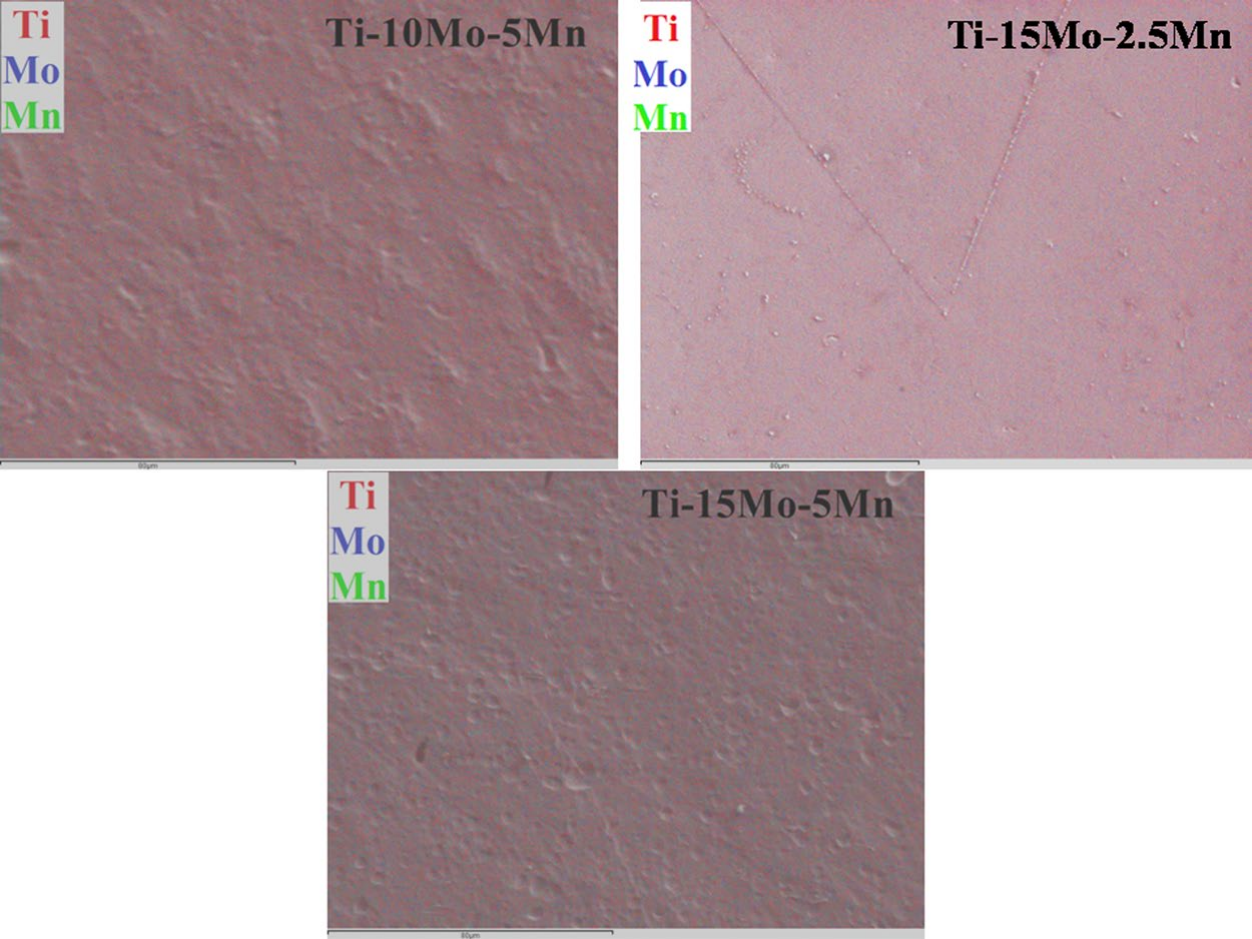
Mapping of alloying elements by energy dispersive spectroscopy (EDS) for Ti-10Mo-5Mn, Ti-15Mo-2.5Mn, and Ti-15Mo-5Mn alloys, where red points represent titanium, blue points represent molybdenum and green points represent manganese.

**Figure 2:**
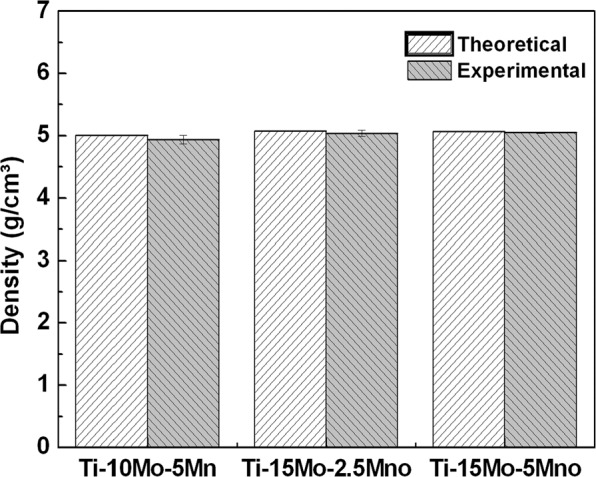
Comparison between theoretical and experimental density of as-cast Ti-10Mo-5Mn, Ti-15Mo-2.5Mn, and Ti-15Mo-5Mn alloys.

The distribution of the alloying elements in the samples was evaluated using EDS, where red points represent titanium, blue points represent molybdenum and green points represent manganese. A good distribution of the elements, without precipitates or aggregates, was observed in all samples (Fig. 1), indicating good homogeneity. The theoretical densities of the samples were compared with the densities obtained by Archimedes’ principle, and these are presented in Fig. 2.

**Figure 3:**
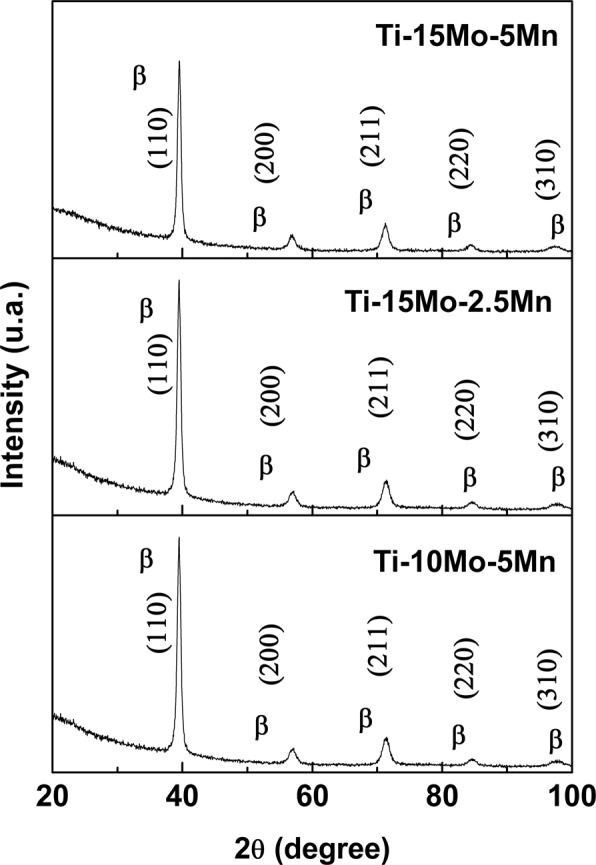
X-ray diffraction patterns of the Ti-10Mo-5Mn, Ti-15Mo-2.5Mn, and Ti-15Mo-5Mn alloys.

Figure 3 shows the x-ray diffraction spectra of the three developed alloys. Only the peaks corresponding to the β-phase of titanium can be observed.

**Figure 4:**
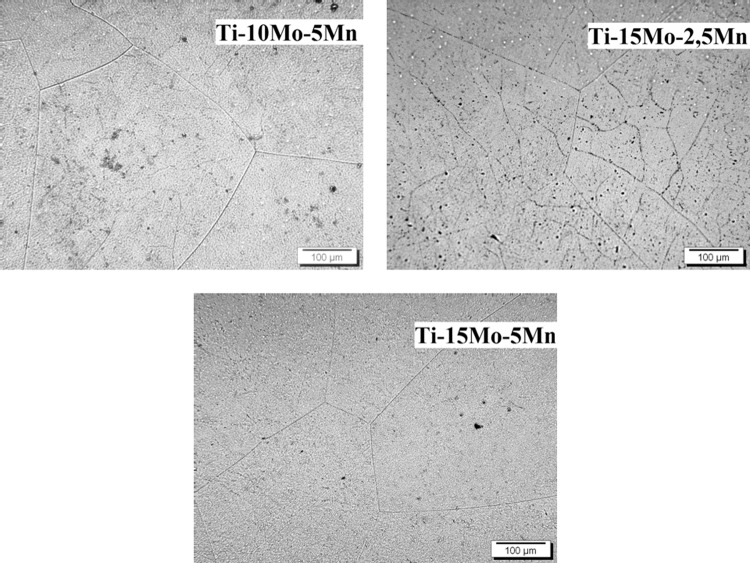
Optical micrographs, with 200x magnification, for Ti-10Mo-5Mn, Ti-15Mo-2.5Mn, and Ti-15Mo-5Mn alloys after melting.

**Figure 5:**
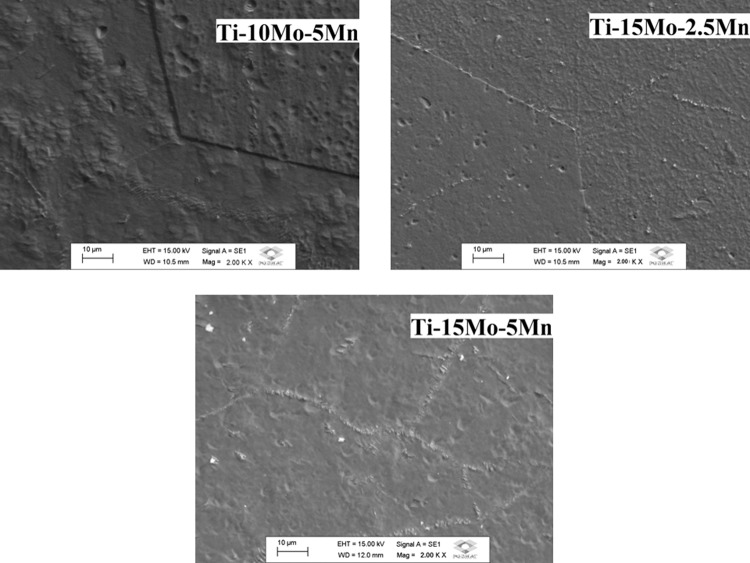
SEM micrographs for as-cast Ti-10Mo-5Mn, Ti-15Mo-2.5Mn, and Ti-15Mo-5Mn alloys, at 2000x magnification.

The optical micrographs for samples of the three prepared alloys are shown in Fig. 4, where the grain boundaries from the β-phase can be clearly observed. In the scanning electron microscope (SEM) micrographs, shown in Fig. 5, only grains from the β-phase can be observed, thus corroborating the optical micrograph results and x-ray diffraction.

**Figure 6:**
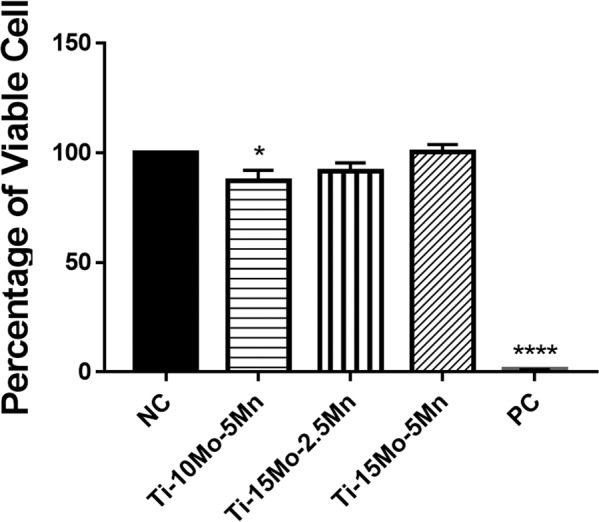
Indirect cytotoxicity test in L929 cells cultured for 48 hours. NC: negative control; PC: positive control.

**Figure 7:**
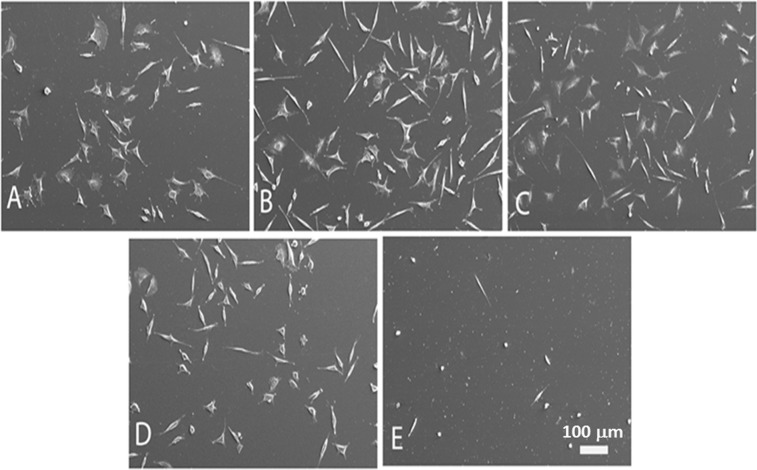
SEM micrographs of the indirect cytotoxicity test in L929 cells cultured for 48 hours. (**A**) Ti-10Mo-5Mn, (**B**) Ti-15Mo-2.5Mn, (**C**) Ti-15Mo-5Mn, (**D**) negative control, and (**E**) positive control.

The results of the indirect cytotoxicity tests for the Ti-10Mo-5Mn, Ti-15Mo-2.5Mn, and Ti-15Mo-5Mn alloys are presented in Fig. 6. The adhesion of the cells is presented in Fig. 7 and shows a central and flattened cell body and numerous and long processes.

## Discussion

Given that the ASTM F2066-13 standard^24^ (which governs the Ti-15Mo alloy) states that the concentration of the elements must be ±1 wt% of the nominal concentration, the alloys of this study are within the established interval. Concerning gas analysis, the results indicated relatively low levels of oxygen and nitrogen present in the alloys, validating the results of the chemical analysis. These concentrations are within the values accepted by ASTM F2066-13 standard, which allows a maximum content of (0.20 ± 0.02) wt% and (0.05 ± 0.02) wt% for oxygen and nitrogen, respectively. Therefore, the interstitials present in the samples are from the melting process and do not compromise the continuation of the study.

A good distribution of the elements, without precipitates, aggregates or agglomerated elements, was observed in all samples, indicating good homogeneity. The experimental values of the density are close to the theoretical values, indicating a good stoichiometry of the samples. The increase in density is related to the density of the elements that compose the alloys. The density of molybdenum (10.28 g/cm³) is greater than that of the other alloying elements, titanium (4.54 g/cm³) and manganese (7.44 g/cm³)^25,26^.

The two elements (molybdenum and manganese) added to titanium are considered β-stabilizers, and previous studies have shown that at least 10 wt% of molybdenum is necessary to have β-phase predominance at room temperature^27^. All the studied alloys satisfy this condition, having 10 wt% and 15 wt% of molybdenum. Therefore, with the addition of manganese, there has been more retention of the β-phase, as shown by the x-ray diffractograms. Other studies have also shown that only the β-phase is present in Ti-xMo alloys with between 15 wt% and 20 wt% of molybdenum^1,28,29^.

In the optical micrographs for samples of the three prepared alloys can be clearly observed the grain boundaries from the β-phase. In the SEM micrographs, only grains from the β-phase can be observed, thus corroborating the optical micrograph and x-ray diffraction results.

Through the microscopy techniques, it was possible to observe distorted and non-uniform grain. This is due to the existence of the temperature gradient during the cooling of the materials after melting, where the bottom of the ingot in contact with the copper (cooled) crucible cools rapidly, while the top of the ingot in contact with the argon atmosphere cools slowly. This gradient results in a nonuniform microstructure. In the future a homogenization heat treatment of the microstructure structure will be performed, the main objective is to recrystallize the material, keeping the β phase as a crystal structure.

The results of the indirect cytotoxicity tests showed that the alloy extracts do not inhibit the proliferation of fibroblast cells, with the number of viable cells being similar to that of the negative control (no cytotoxicity) and higher than that of the positive control (cytotoxicity: phenol solution). These differences were not statistically significant. Cell viability with the Ti-10Mo-5Mn alloy extract was significantly lower compared to the negative control (p < 0.05) but higher compared to the positive control (cytotoxicity: phenol solution). The Ti-15Mo-2.5Mn and Ti-15Mo-5Mn alloy extracts showed no statistically significant difference (p = 0.05). However, none of the alloy extracts inhibited the proliferation of fibroblast cells and the number of viable cells when compared with the positive control (cytotoxicity). The number of viable cells for the positive control was significantly less than that of the negative control (p < 0.0001). Similar to the negative control, none of the studied alloy extracts changed the cell morphology. The cells were observed to have well adhered as were the cells treated with the medium base (negative control). All of them, regardless of the alloy extract, showed a central and flattened cell body and numerous and long processes.

In conclusion, Ti-Mo-Mn alloys were produced by arc-furnace melting. The chemical compositions of the samples were close to the nominal values, with no significant presence of other metallic impurities and with low gas contents. The alloy densities confirmed the good stoichiometry of the ingots. The alloy structures and microstructures showed only the β-phase in the form of equiaxial grains. The studied alloy extracts were not cytotoxic for fibroblast cells in short culture periods.

## Methods

For the manufacture of the alloys, commercially pure precursors were used. They were properly separated and cleaned. The titanium and molybdenum were cleaned in an acidic solution (40 ml of HNO_3_ and 10 ml of HF). The manganese was cleaned in a Keller solution (50 ml of H_2_O, 25 ml of HNO_3_, 15 ml HCl, and 10 ml of HF).

Ti-10Mo-5Mn, Ti-15Mo-2.5Mn and Ti-15Mo-5Mn alloys were melted in an arc-furnace with water-cooled copper crucible and argon inert atmosphere (1 atm). The arc melting technique is widely used to produce high melting titanium alloys. Several works in the literature use this technique to produce metallic alloys^30–32^. In these works alloys with the tantalum elements in the chemical composition, melting point exceeding 3,273 K are satisfactorily produced^33–35^.

The melt was performed with the precursors inserted in the crucible, and after the furnace closed, approximately 10^−2^ Torr vacuum was made for 30 minutes, followed by a purging process, inserting argon, which is an inert gas, maintaining for 3 minutes, and subsequently subjected to a new vacuum cleaning and maintained for 10 minutes. This procedure was performed five times in each sample melting to ensure a good purity of the alloys. The ingots weighed approximately 60 g.

It is necessary to perform analyses to know the chemical concentration of the elements and possible impurities present in the alloy, to have the guaranty that in the preparation process there were no changes in the quantity of the elements or addition of impurities, causing changes in the final constitution of the alloy.

The chemical composition of the alloys was analyzed using ICP-OES equipment (Varian, Vista model). For the mapping, EDS was used (Oxford, INCA x-act detector), coupled to a scanning electron microscope. Density measurements were obtained using Archimedes’ principle. The gas analysis was conducted by absorption of infrared radiation in a nitrogen/oxygen determinator LECO TC-400 equipment.

Structural analysis was performed by means of x-ray diffraction measurements using a Rigaku MiniFlex600 diffractometer with Cu Kα radiation (λ = 1.54056 Å). The data were collected using the powder method and the fixed-time mode, with steps of 0.04°, ranging from 20° to 100°, with 2θ step and 10°/min collection time.

Microstructural analysis was performed with an Olympus, BX51M model optical microscope, which was performed with 200x magnification micrographs and a Carl Zeiss scanning electron microscope, EVO LS15 model, with 2000x magnification.

For the cytotoxic tests, L929 mouse fibroblast cells were maintained in Dulbecco modified Eagle medium (DMEM) supplemented with 10% fetal bovine serum (FBS). The cells were incubated under standard cell culture conditions (37 °C, 95% humidity and 5% CO_2_) and the medium was changed every two to three days. The alloy extracts were prepared with 1 g of alloy per 1 mL of DMEM at 37 °C for 48 hours. The indirect cytotoxicity of the Ti-10Mo-5Mn, Ti-15Mo-2.5Mn and Ti-15Mo-5Mn alloys were determined using the MTT assay for 48 hours, as described in other studies^36,37^. The cells were cultivated in 96-well microplates and incubated at a density of 110 cells/mm². The medium base was used as the negative control (no cytotoxicity) and was substituted by the obtained alloy extract, while a solution of DMEM, 10% FBS, and 1% phenol was used as the positive control (cytotoxicity). The optical density (OD) of the wells was determined using a plate reader at a test wavelength of 640 ηm in a SpectraMax Plus Microplate Reader (Molecular Devices). In the SEM analysis, fibroblast cells were put on the glass coverslip and the culture medium was substituted by the obtained alloy extracts. After 48 hours, the cells were fixed and were then routinely processed for SEM analysis^38^. Specimens were mounted onto aluminum substrates, sputtered with gold, and examined in a scanning electron microscope (Carl Zeiss EVO LS15). Polystyrene was used as the negative control (no cytotoxicity), while a solution of α-MEM, 10% FBS, and 1% phenol was a positive control (cytotoxicity). The results were analyzed statistically by a t-test. The level of significance was 5% (p ≤ 0.05).

## References

[1] Correa DRN, Kuroda PAB, Grandini CR (2014). Structure, Microstructure, and Selected Mechanical Properties of Ti-Zr-Mo Alloys for Biomedical Applications. Advanced Materials Research.

[2] Xavier, C. C., Correa, D. R. N., Grandini, C. R. & Rocha, L. A. Low temperature heat treatments on Ti-15Zr-xMo alloys. *Journal of Alloys and Compounds* (2017).

[3] Geetha M, Singh AK, Asokamani R, Gogia AK (2009). Ti based biomaterials, the ultimate choice for orthopaedic implants – A review. Progress in Materials Science.

[4] Niinomi M (2008). Mechanical biocompatibilities of titanium alloys for biomedical applications. Journal of the mechanical behavior of biomedical materials.

[5] Niinomi M (2002). Recent Metallic Materials for Biomedical Applications. Metall and Mat Trans A.

[6] Cordeiro JM, Barão VA (2017). Is there scientific evidence favoring the substitution of commercially pure titanium with titanium alloys for the manufacture of dental implants?. Materials Science and Engineering: C.

[7] Domingo JL (2002). Vanadium and tungsten derivatives as antidiabetic agents. Biological trace element research.

[8] Sigel, H. *Metal ions in biological systems: volume 37: manganese and its role in biological processes*. (CRC press, 2000).

[9] Chen Q, Thouas GA (2015). Metallic implant biomaterials. Materials Science and Engineering: R: Reports.

[10] Santos PF (2016). Fabrication of low-cost beta-type Ti–Mn alloys for biomedical applications by metal injection molding process and their mechanical properties. Journal of the Mechanical Behavior of Biomedical Materials.

[11] Chui P (2017). Near β-type Zr-Nb-Ti biomedical alloys with high strength and low modulus. Vacuum.

[12] Niinomi M, Liu Y, Nakai M, Liu H, Li H (2016). Biomedical titanium alloys with Young’s moduli close to that of cortical bone. Regenerative biomaterials.

[13] Wong, J. Y. & Bronzino, J. D. (Taylor & Francis group, Boca Raton, London, New York, 2007).

[14] Park, J. & Lakes, R. S. In *Biomaterials* 99–137 (Springer New York, 2007).

[15] Ho WF, Ju CP, Chern Lin JH (1999). Structure and properties of cast binary Ti–Mo alloys. Biomaterials.

[16] Hong SH, Park SW, Park CH, Yeom J-T, Kim KB (2020). Relationship between phase stability and mechanical properties on near/metastable β-type Ti–Cr-(Mn) cast alloys. Journal of Alloys and Compounds.

[17] Kang N, Li Y, Lin X, Feng E, Huang W (2019). Microstructure and tensile properties of Ti-Mo alloys manufactured via using laser powder bed fusion. Journal of Alloys and Compounds.

[18] Correa DRN (2015). Effect of the substitutional elements on the microstructure of the Ti-15Mo-Zr and Ti-15Zr-Mo systems alloys. Journal of Materials Research and Technology.

[19] Martins, J. R. S., Araújo, R. O., Nogueira, R. A. & Grandini, C. R. Internal Friction and Microstructure of Ti and Ti-Mo Alloys Containing Oxygen. *Archives of Metallurgy and Materials* **61**, 10.1515/amm-2016-0011 (2016).

[20] Liu X, Chen S, Tsoi JKH, Matinlinna JP (2017). Binary titanium alloys as dental implant materials—a review. Regenerative Biomaterials.

[21] Alrabeah GO, Brett P, Knowles JC, Petridis H (2017). The effect of metal ions released from different dental implant-abutment couples on osteoblast function and secretion of bone resorbing mediators. Journal of dentistry.

[22] Miao L, Clair DKS (2009). Regulation of superoxide dismutase genes: implications in disease. Free Radical Biology and Medicine.

[23] Santos PF (2015). Microstructures, mechanical properties and cytotoxicity of low cost beta Ti–Mn alloys for biomedical applications. Acta biomaterialia.

[24] ASTM. in *F2066-08 - Standard specification for wrought titanium-15 molybdenum alloy for surgical implant application* Vol. F 2066-08 (ASTM International, Philadelphia (USA), 2008).

[25] Kuroda PAB, Buzalaf MAR, Grandini CR (2016). Effect of molybdenum on structure, microstructure and mechanical properties of biomedical Ti-20Zr-Mo alloys. Materials Science and Engineering: C.

[26] Lide, D. *CRC handbook of chemistry and physics: a ready-reference book of chemical and physical data*. 85th edn, (CRC Press, 2004).

[27] Bania P (1994). Beta titanium alloys and their role in the titanium industry. JOM.

[28] Oliveira NTC, Aleixo G, Caram R, Guastaldi AC (2007). Development of Ti–Mo alloys for biomedical applications: Microstructure and electrochemical characterization. Materials Science and Engineering: A.

[29] Martins Júnior JRS (2011). Preparation and characterization of Ti-15Mo alloy used as biomaterial. Materials Research.

[30] Correa DRN (2018). Development of Ti-15Zr-Mo alloys for applying as implantable biomedical devices. Journal of Alloys and Compounds.

[31] Correa DRN, Kuroda PAB, Lourenço ML, Buzalaf MAR, Grandini CR (2019). Adjustment of the microstructure and selected mechanical properties of biomedical Ti-15Zr-Mo alloys through oxygen doping. Journal of Alloys and Compounds.

[32] Pintão CAF, Correa DRN, Grandini CR (2019). Torsion modulus as a tool to evaluate the role of thermo-mechanical treatment and composition of dental Ti-Zr alloys. Journal of Materials Research and Technology.

[33] Kuroda PAB, Lourenço ML, Correa DRN, Grandini CR (2020). Thermomechanical treatments influence on the phase composition, microstructure, and selected mechanical properties of Ti–20Zr–Mo alloys system for biomedical applications. Journal of Alloys and Compounds.

[34] Quadros FDF, Kuroda PAB, Sousa KDSJ, Donato TAG, Grandini CR (2019). Preparation, structural and microstructural characterization of Ti-25Ta-10Zr alloy for biomedical applications. Journal of Materials Research and Technology.

[35] Kuroda PAB, Quadros FDF, Araújo ROD, Afonso CRM, Grandini CR (2019). Effect of Thermomechanical Treatments on the Phases, Microstructure, Microhardness and Young’s Modulus of Ti-25Ta-Zr Alloys. Materials.

[36] Donato TAG (2009). Cytotoxicity study of some Ti alloys used as biomaterial. Materials Science and Engineering: C.

[37] Mosmann T (1983). Rapid colorimetric assay for cellular growth and survival: Application to proliferation and cytotoxicity assays. Journal of Immunological Methods.

[38] Donato T, de Almeida L, Arana-Chavez V, Grandini C (2014). *In Vitro* Cytotoxicity of a Ti-35Nb-7Zr-5Ta Alloy Doped with Different Oxygen Contents. Materials.

